# Single-cell analysis reveals differences among *i*NKT cells colonizing peripheral organs and identifies *Klf2* as a key gene for *i*NKT emigration

**DOI:** 10.1038/s41421-022-00432-z

**Published:** 2022-08-02

**Authors:** Jie Wang, Ian Loveless, Indra Adrianto, Tingting Liu, Kalpana Subedi, Xiaojun Wu, Md Moazzem Hossain, Eric Sebzda, Li Zhou, Qing-Sheng Mi

**Affiliations:** 1 Center for Cutaneous Biology and Immunology, Department of Dermatology, Henry Ford Health, Detroit, MI USA; 2 Immunology Research Program, Henry Ford Cancer Institute, Henry Ford Health, Detroit, MI USA; 3 Center for Bioinformatics, Department of Public Health Sciences, Henry Ford Health, Detroit, MI USA; 4 Department of Biochemistry, Microbiology, and Immunology, Wayne State University School of Medicine, Detroit, MI USA

**Keywords:** Immunology, Cell biology

## Abstract

Invariant natural killer T cell (*i*NKT) subsets are differentially distributed in various immune organs. However, it remains unclear whether *i*NKT cells exhibit phenotypical and functional differences in different peripheral organs and how thymic *i*NKT cells emigrate to peripheral organs. Here, we used single-cell RNA-seq to map *i*NKT cells from peripheral organs. *i*NKT1 cells from liver, spleen, and lymph node appear to have distinct phenotypic profiles and functional capabilities. However, *i*NKT17 transcriptomes were comparable across peripheral organs. In addition, by integrating data with a thymic *i*NKT cell study, we uncovered a transient population of recent thymic emigrants, a cluster of peripheral *i*NKT cells with high expression of transcription factor Kruppel-like factor 2 (*Klf2*). Deletion of *Klf2* led to a severe impairment of *i*NKT differentiation and migration. Our study revealed that *i*NKT subsets are uniquely distributed in peripheral organs with some inter-local tissue variation, especially for *i*NKT1 cell, and identified *Klf2* as a rheostat for *i*NKT cell migration and differentiation.

## Introduction

Invariant natural killer T (*i*NKT) cells are innate-like T cells that are initially selected by CD1d and preferentially use an invariant T cell receptor (TCR) consisting predominantly of the Vα14-Jα18/Vβ8 pair in mice^1–3^. *i*NKT cells were known to develop in the thymus through a four-stage process in mice: stage 0 (CD24^+^), stage 1 (CD24^−^CD44^−^NK1.1^−^), stage 2 (CD44^+^NK1.1^−^), and stage 3 (CD44^+^NK1.1^+^). During development, thymic *i*NKT cells display substantial functional heterogeneity, with three major subsets, including *i*NKT1, *i*NKT2, and *i*NKT17. These three major functional subsets exhibit distinct transcription factors and cytokine production. For instance, *i*NKT1 cells are T-bet^+^ and mainly produce IFN-γ; *i*NKT2 cells are PLZF^hi^ and mainly produce IL-4; and *i*NKT17 cells are RORγt^+^ and produce IL-17. *i*NKT1 cells develop through all stages and finally mature in stage 3, which also highly express cytolytic effectors (perforin, granzyme B, granzyme A, and FAS ligand) and specific chemokines and their receptors. However, *i*NKT2 and *i*NKT17 cells terminate at stage 2. Stage 2 *i*NKT cells have a high proliferation capability, and most *i*NKT cells at this stage emigrate to peripheral organs^4^.

After emigrating from the thymus, *i*NKT cells are differentially distributed in various peripheral organs. For instance, only a small subset of *i*NKT cells traffic through the lymph nodes (0.2%–1%), with the largest *i*NKT cell populations localizing to the liver (12%–30%), lung (5%–10%), and spleen (1%–3%)^5–7^. Most *i*NKT cells are tissue-resident and noncirculating^4,8^, with *i*NKT1 cells being dominant in the liver, while lymph node shows enrichment for *i*NKT17 cells, and spleen and lungs show preference to *i*NKT2 cells. This is thought to be mediated by the differences in chemokine receptor expression profiles in *i*NKT cells and tissue microenvironment^9–11^. Many studies have demonstrated that *i*NKT cells play a critical role in various pathological conditions, including cancer, autoimmune disease, and infection, and *i*NKT cells from different organs appear to have distinct functional capabilities^2,12,13^. More specifically, recent studies suggest that *i*NKT cells from spleen, thymus, and liver exhibit different anti-tumor activity in varying tumor models^9,14^, including MCA-1 sarcoma and B16F10 melanoma metastasis models. However, the underlying mechanism of *i*NKT functional differences in different organs is still unclear.

*i*NKT cell emigration from the thymus to peripheral organs mainly occurs at stage 2, in a *Ccr7*-dependent fashion. However, *Ccr7* is also a lineage-defining marker for *i*NKT multipotent precursors^15^, which makes studying recent thymic emigrants (RTE) difficult. In addition, RTEs are a rare population relative to total peripheral iNKT cells and thus the mechanisms responsible for *i*NKT thymic emigration and *i*NKT cell distribution in peripheral organs remain unknown.

Several pioneering studies, including our own, have indicated that thymic *i*NKT cells are more plastic than their defined *i*NKT1/2/17 sublineages^16^. However, whether the thymic *i*NKT clusters are conserved in peripheral organs is still not quite clear. In this study, we extended our analysis of subsets of *i*NKT cells in peripheral organs. We performed single-cell RNA-sequencing (scRNA-seq) of *i*NKT cells from liver, spleen, and lymph node and revisited our scRNA-seq of thymic *i*NKT cells. We found that there are substantial differences between *i*NKT cells in the thymus and in peripheral organs. More importantly, in peripheral organs, *i*NKT cells from liver and spleen showed great commonality, but *i*NKT cells from lymph node showed great phenotypical differences with *i*NKT from either liver or spleen, especially for *i*NKT1 cells. Via integration with thymic *i*NKT cells, we identified an RTE cluster among peripheral *i*NKT cells, which highly express *Klf2*. Studies from *Klf2* deletion mouse models indicated that *Klf2* is a key regulator for *i*NKT cell differentiation and migration. Taken together, our data constitute a comprehensive analysis of the similarities and differences among functionally distinct peripheral *i*NKT cells and provide a valuable resource for future disease model studies.

## Results

### Overview of the cell types in peripheral *i*NKT cells identified by scRNA-seq

**Fig. 1:**
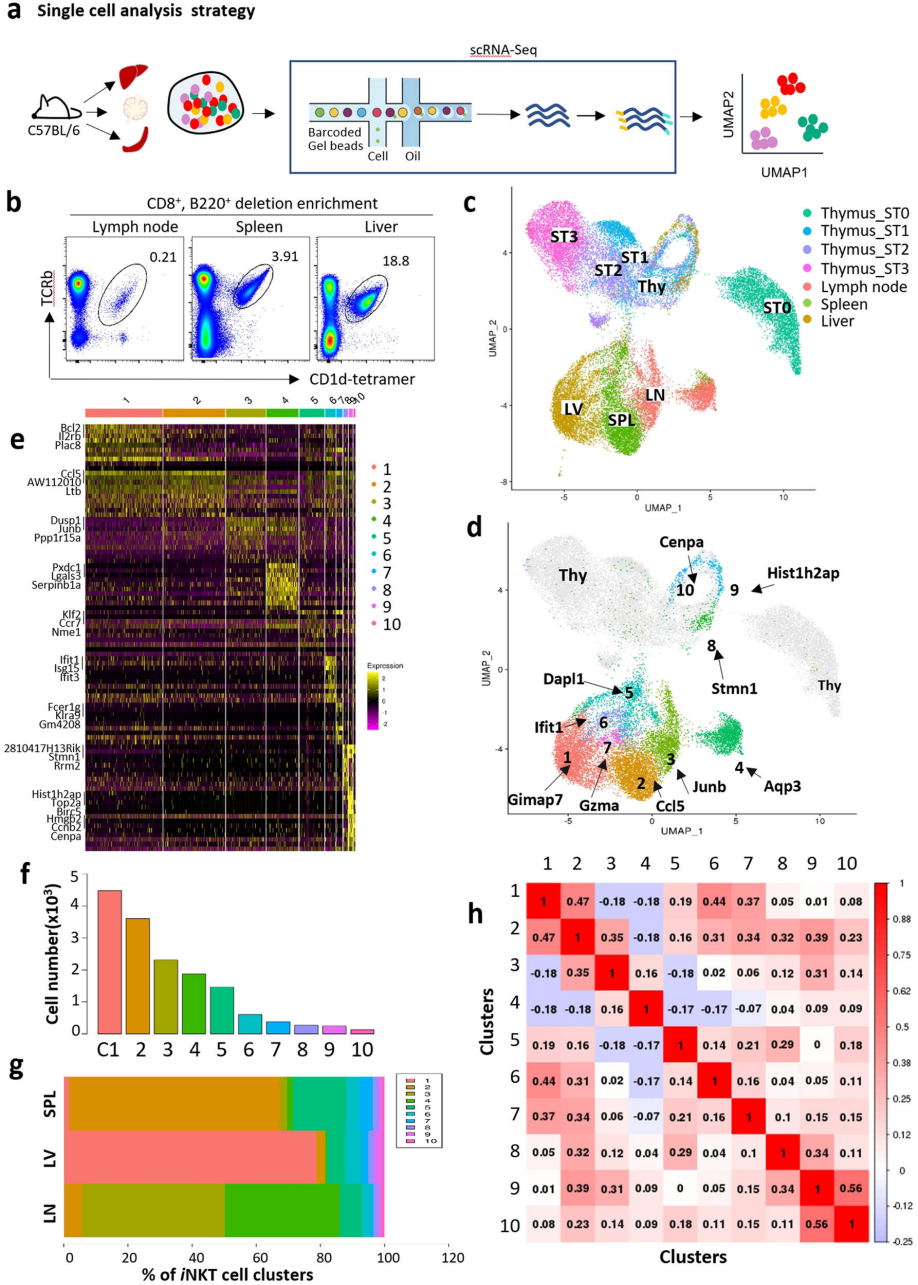
The diversity of mouse peripheral *i*NKT cells. **a** *i*NKT cells collected from peripheral organs including liver, spleen, and lymph node for scRNA-Seq analysis. **b** Sorting strategy of *i*NKT cells from liver, spleen, and lymph node post CD8^+^B220^+^ deletion enrichment. **c** UMAP plots from 10X genomics scRNA-Seq dataset from sorted peripheral *i*NKT cells along with thymic different stages of *i*NKT cells collected. Displaying relationships between *i*NKT cell organs origins. **d** UMAP plots of data identical to those in **c**, but color coded on *i*NKT cell clusters. **e** Heatmap of the top ten differentially regulated genes from each cluster derived from **d**. Each column represents gene expression for an individual cell with color coded on gene expression profiles. Yellow is upregulated and purple is downregulated. **f** Bar graph showing the cell number in each of clusters. **g** The fractions of ten clusters defined in peripheral *i*NKT cells. **h** Pearson correlation matrix of the average expression profiles based on all differentially expressed genes from all clusters analyzed.

We previously studied cellular heterogeneity of mouse thymic *i*NKT cells using scRNA-seq. To capture the extent of cellular heterogeneity within mouse peripheral *i*NKT cells, we applied scRNA-seq (10X genomics chromium) on *i*NKT cells from six samples representing three peripheral tissues, including spleen, liver, and lymph node (Fig. 1a, b). After quality control (Supplementary Fig. S1a, b), a total of 5570 individual *i*NKT cells (sample #1: 2760; sample #2: 2810) from liver; 4850 individual *i*NKT cells (sample #1: 2562; sample #2: 2288) from spleen; and 4897 individual *i*NKT cells (sample #1: 2408; sample #2: 2489) from lymph node were assessed for single-cell RNA expression. *i*NKT cells from three organs that we profiled in replicates were well correlated (Spearman’s coefficient: 0.92 between liver *i*NKT cells; 0.89 between splenic *i*NKT cells, and 0.91 between lymph node *i*NKT cells) (Supplementary Fig. S1c).

After processing the sequencing data using the Cell Ranger pipeline (10X Genomics), we performed unbiased clustering analysis of peripheral *i*NKT cells with thymic *i*NKT cells (over 17,000 cells) using Seurat. *i*NKT cells from peripheral organs cluster uniquely from thymic *i*NKT cells (Fig. 1c), reflecting substantially different transcription programs between thymic *i*NKT cells and peripheral *i*NKT cells. Among integrated peripheral *i*NKT cells, a total of 10 clusters (C1–C10) were identified (Fig. 1c–e), with as few as 123 cells to as many as 4474 cells per cluster (Fig. 1f, g), with some clusters (C4–C10) shared between *i*NKT cells from different organs.

We used differential gene expression analysis to determine cell type-specific marker genes with highly different transcriptional levels between clusters. The top 10 genes from individual clusters were shown in heatmap (Fig. 1e). Correlation analysis of marker gene signatures revealed that similar cell types clustered together (Supplementary Fig. S1d). For initial analysis, we found that most *i*NKT cells from liver, spleen, and lymph node were clearly separated, indicating that tissue location is associated with specific transcriptomic program in these *i*NKT cells (Fig. 1c), e.g., cluster C1 from liver, C2 from spleen, and C3 from lymph node (Fig. 1d), and the similarity of different clusters from different organs were shown in Fig. 1h. These clusters were recognized as main *i*NKT clusters with natural killer properties; however, we found that the C4–C10 clusters were mixed across all samples (Fig. 1c, d), independent of their location nature. These data suggest that an intrinsic *i*NKT program is the main determinant of the transcriptional profile of C4–C10 *i*NKT cells. *i*NKT cells from lymph node made a major contribution to C4, which highly expressed *i*NKT17 signature genes, including *Rorc*, *Pxdc1*, and *Aqp3*. Cluster C5 shows high levels of *Ccr7* and *S1pr1* that are associated with cell homing. C6 shows high levels of *Ifit1* and *Ifit3*. Given that *Ifit1*/*3* function as inhibiting viral replication and translational initiation, we assumed that C6 *i*NKT cells may be associated with anti-viral function. C7 shows high expression level of *Gzma*, which is associated with cellular cytotoxic properties. C8–C9 show a great overlap with thymic stage 1 and 2 *i*NKT cells with high expression of genes regulating the cell cycle (discussed below). Clusters identified in mRNA levels were also verified with available antibodies at protein levels. As shown in Supplementary Fig. S2a–c, RORγt^+^ *i*NKT17 cells (C4) in different peripheral organs showed a dramatical increased Aqp3 expression, while IFIT1 (C6) had an increased expression pattern as compared with conventional T cells in different peripheral organs. In addition, Gzma (C7) was identified in *i*NKT cells from lymph nodes, spleen, and liver, with varying expression enrichment, especially in NK1.1^+^ mature *i*NKT cells.

### Defining the distinct subsets of *i*NKT cells in peripheral organs

**Fig. 2:**
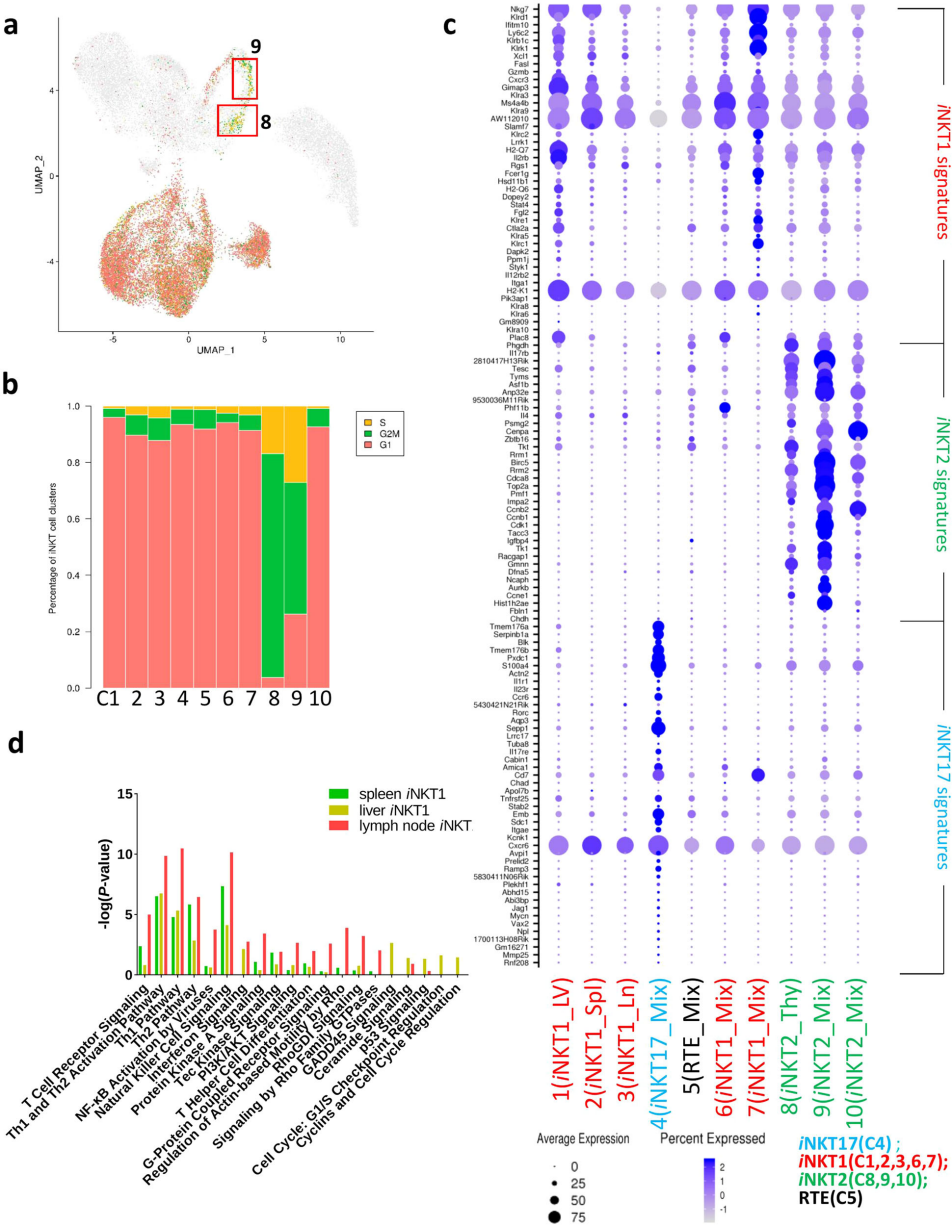
Different clusters assigned into functional subsets. **a** UMAP plots depicting single-cell genes expression trajectory of G1, G2/M, and S phages in peripheral *i*NKT cell development. **b** Bar graph represents fraction of G1, G2/M, and S cells in *i*NKT cells clusters. **c** Bubble plots showing gene expression in individual clusters (C1–C10) from aggregated *i*NKT cells. Gene names labeled in blue are *i*NKT17 signature genes, in red are *i*NKT1 signature genes and in green are *i*NKT2 signature genes. X axis shows different clusters identified in Fig. 1d. **d** Bar graph showing IPA analysis of *i*NKT1 cells in different peripheral organs.

To explore *i*NKT cell diversity in more detail, we annotated 10 clusters from peripheral organs with assumed cell-type identities based on known marker genes derived from published expert annotation^16^. Among these 10 peripheral *i*NKT cell clusters, we noticed that clusters C8–C9 were mainly proliferating cells showing high expression levels of S and G2M cell-cycle marker genes (Fig. 2a, b; Supplementary Fig. S3a). To avoid the possibility that proliferating genes were affecting cellular cluster analysis, we isolated these clusters and corrected the gene expression levels for cell cycle phase. Subsequent unsupervised clustering analysis revealed that cell clusters were similar to those in primary data (data not shown), indicating that cell-cycle genes did not disturb cell cluster analysis in this study.

*i*NKT1/2/17 subsets have been identified based on their transcription factor and cytokine production profiles. Although these differentiated *i*NKT subsets initially emerge in the thymus, it is likely that tissue-specific factors and local environmental influences shape the phenotype and function after recruitment to specific tissue sites. Therefore, we dissected peripheral *i*NKT cells into transcriptionally distinct subpopulations, which could allow us to access the transcriptional landscape in a more detailed *i*NKT functional model.

Based on published lineage markers of *i*NKT cells lineages^16^, we found that clusters C1–C3, clusters C6 (*Ifit1*/*Ifit3*), and C7 (*Gzma*) were categorized into *i*NKT1 subsets. These cells expressed *Ifng* and various NK cell lectin-like receptors, such as *Klra1*, *Klrb1*, and *Klrc2*, which may regulate cytotoxic properties. In this study, most *i*NKT1 cells from different organs were not clustered together, e.g., C1 from liver, C2 from spleen, and C3 from lymph node, indicating that *i*NKT1 cells exhibit tissue-specific variations in phenotype; clusters C8–C10 from three peripheral organs show highly expressing Gata3, Icos, and Izumol1r (Supplementary Fig. S3b), were assigned as *i*NKT2 cells; cluster C4, mainly from lymph node, and a small fraction from spleen and liver, were categorized into *i*NKT17 subset, expressing high levels of *Rorc*, *Il17rb*, *Il23r*, *Tmem176b*, *Ccr6*, and *Ccr8* (Fig. 2c). Given that *i*NKT17 cells from all different peripheral organs cluster together as single C4, this suggests that unlike *i*NKT1 cells, *i*NKT17 transcriptional programs might not be dependent on specific tissue environment. Cells in C5 had a high expression of the homing marker, *Ccr7* (Fig. 1e). Interestingly, this cluster did not stand out in any *i*NKT subsets, showing an undifferentiated status that could represent RTE *i*NKT cells. In addition, we found that *i*NKT17 cells were identical across different peripheral organs and *i*NKT2 cells showed proliferative activity. However, *i*NKT1 cells differ from organ to organ. Ingenuity pathway analysis (IPA) data indicated that *i*NKT1 from lymph nodes enriched in T cell receptor signaling, Tec Kinase signaling, and T helper cell differentiation pathways. *i*NKT1 from liver showed a higher cellular proliferation status (Fig. 2d).

### Comparison of transcriptomic profiles among *i*NKT cells from different peripheral organs

**Fig. 3:**
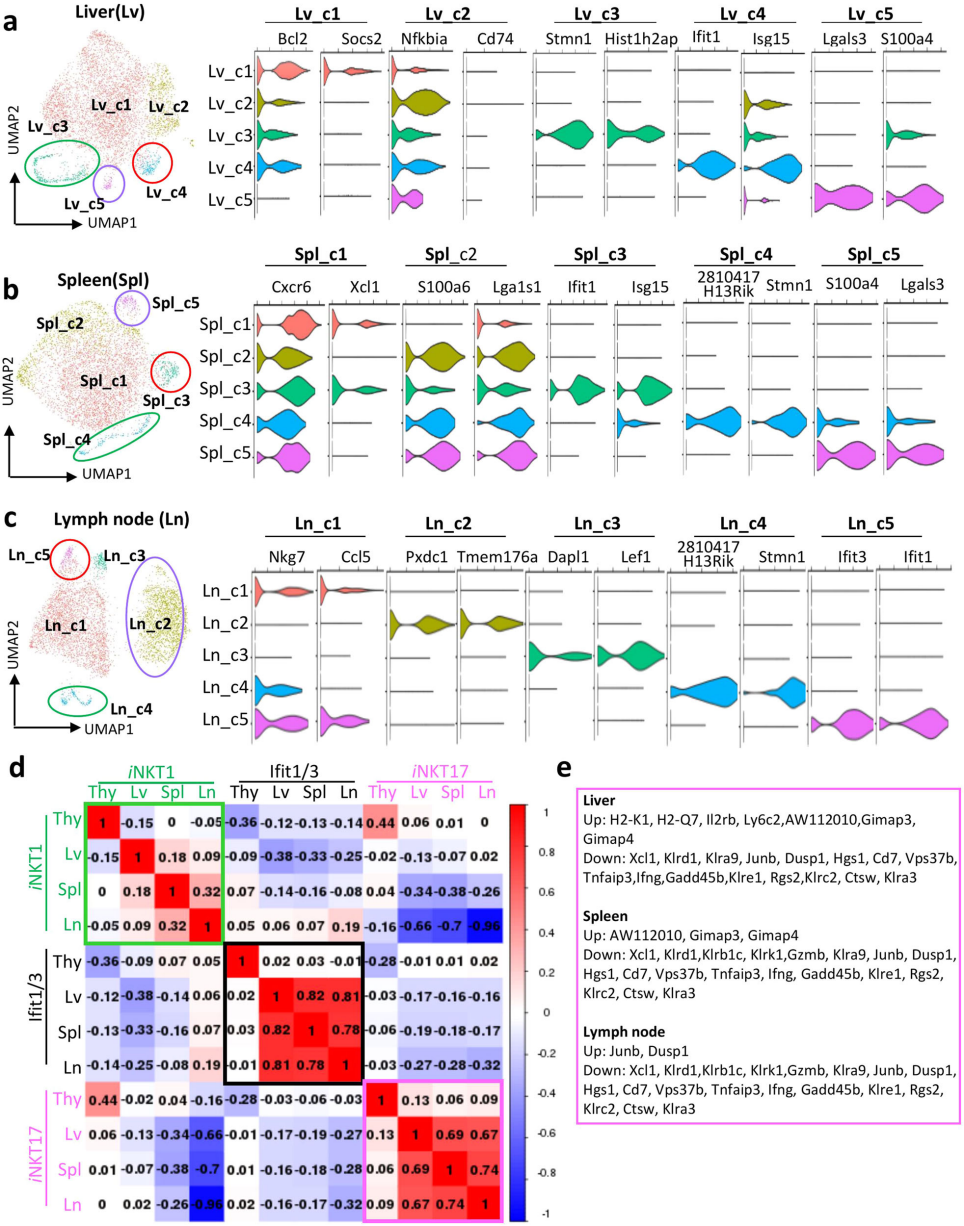
Comparison of transcriptomic profiles among *i*NKT cells from different peripheral organs. **a**–**c** UMAP plots from 10X genomics scRNA-Seq dataset from sorted liver *i*NKT cell(a), spleen *i*NKT cell (**b**), and Lymph node *i*NKT cells (**c**) (left). Violin plots showing the top two cluster-specific signatures in *i*NKT cells from liver (**a**), spleen (**b**), and lymph node (**c**). **d** Pearson correlation matrix of the average expression profiles based upon all differentially expressed genes from all indicated subpopulations analyzed. **e** Gene list showing significant change of *i*NKT1 signatures among *i*NKT cells from thymus, liver, spleen, and lymph node, extracted from (**d**).

*i*NKT cells in different lymphoid organs display distinct tissue tropisms, which might be mediated by local environment. Previous studies have indicated that *i*NKT cells from different organs mediate a different functional capability^2,9,14^. Therefore, we compared *i*NKT cells from different peripheral organs. As shown in Fig. 3a–c, we detected five distinct clusters in peripheral *i*NKT cells from liver, spleen, and lymph node. Violin plots with the indicated UMAP plot show the top two signatures of each cluster in indicated peripheral organs; the top 10 signatures for each cluster were provided in Supplementary Fig. S4a–c. *i*NKT1 clusters found in all three organs (Lv_C1 in liver, Spl_C1 in spleen, and Ln_C1 in lymph node) were all assigned to *i*NKT1, but with different transcriptomic profiles. Ln_C5 in lymph node, Spl_C3 in spleen, and Lv_C4 in liver were identified as an *Ifit1/3* cluster, which also showed *i*NKT1 characteristics; Ln_C4 in lymph node, Spl_C4 in spleen, and Lv_C3 in liver were cell cycle clusters; Ln_C2 in lymph node, Spl_C5 in spleen, and Lv_C5 in liver were identified as *i*NKT17 cells. In addition, we observed that Lv_C2 with *Gadd45b*, *Icam1*, *Nfkbia*, *Irf8*, *Relb*, and *Ifng* expression was found only in liver (Supplementary Fig. S4d). Further signaling pathway analysis indicated that besides NK cell-mediated cytotoxicity pathway, this cluster was much more enriched in NF-κB signaling pathway and T cell receptor signaling pathway (Supplementary Fig. S4e). This result was initially curious to us, because T cell-NF-κB is important for IFN-γ production and plays an important role in anti-tumor immunity^17^.

To test whether *i*NKT cells in the thymus and in different peripheral organs display similar genomic profiles, we re-visited our previous single-cell study of thymic *i*NKT cells (data not published). With these data, we carried out a scRNA-seq-based comparison between assigned clusters among *i*NKT cells from indicated organs (Fig. 3d). These data reflected the extensive transcriptomic differences between *i*NKT cells from thymus, liver, spleen, and lymph node. There was less overlap of transcriptomic expression between *i*NKT1 from thymus and peripheral organs (Lv_C1, Spl_C1, and Ln_C1), where the Silhouette coefficient was −0.15 for liver *i*NKT1 cells, 0 for spleen *i*NKT1 cells, and −0.05 for lymph node *i*NKT1 cells relative to thymic *i*NKT1 cells (Fig. 3d). *i*NKT1 cells were also shown to be tissue-specific in peripheral organs, where the Spearman’s coefficient was 0.13 between liver and spleen, 0.32 between spleen and lymph node, and 0.09 between liver and lymph node (Fig. 3d). Interestingly, we observed that anti-apoptotic gene *Bcl2* and suppressor of cytokine signaling 2 (*Socs2)* were highly expressed in *i*NKT cells from liver, but not in *i*NKT cells from spleen and lymph node (Supplementary Fig. S5a). The differences in *Bcl2* and *Socs2* expression by *i*NKT cells in different organs could explain the enrichment of *i*NKT cell population in liver. Given that the *Ifit1/3* cluster was identified in all organs, we explored their similarity among organs, and we observed a strong correspondence of *Ifit1/3* clusters among peripheral organs (Spearman’s coefficient was 0.82 between liver and spleen, 0.78 between spleen and lymph node, and 0.81 between liver and lymph node). However, the Spearman’s coefficient between *i*NKT cells from the thymus and peripheral organs varied from −0.01 to 0.02 (Fig. 3d). A similar phenomenon was also observed in *i*NKT17 clusters, namely, a high correlation in *i*NKT17 among peripheral organs (Spearman’s coefficient is 0.69 between liver and spleen; 0.74 between spleen and lymph node; and 0.67 between liver and lymph node); however, the coefficient between thymic *i*NKT17 cells and peripheral *i*NKT17 varied from −0.06 to 0.013 (Fig. 3d). These data indicate that *i*NKT17 cells exhibit a nearly identical transcriptome in peripheral organs.

Comparison of populations in heatmaps is provided in Supplementary Fig. S5b–d. These analyses again highlighted many similarities between *i*NKT cells from liver, spleen, and lymph node, and showed how transcriptomes change when *i*NKT cells emigrate from the thymus to different peripheral organs. This includes genes that were differentially expressed between *i*NKT1 cells from the liver vs the thymus (Fig. 3e; Supplementary Fig. S5b). *i*NKT17 cells show a lower expression ratio of *Furin*, *Emb*, *Rorc*, *Il17a*, *Avpl1*, and *Ly6a* in peripheral organs, as compared with those from the thymus. However, there was no marked difference in transcriptomic profiles of *i*NKT17 cells in different peripheral organs (Supplementary Fig. S5c). In addition, the *Ifit1/3* cluster from peripheral organs exhibits a great similarity between different organs, but they were more pronounced in *i*NKT cells from peripheral organs (Fig. 3d; Supplementary Fig. S5d). Overall, these data indicate that *i*NKT cells from the thymus exhibit a great transcriptomic difference from those in peripheral organs, and transcriptomic profiles of *i*NKT1 cells correlated poorly within different peripheral organs; however, the transcriptional patterns were highly correlated for *i*NKT17 cells and the *Ifit1/3* cluster among peripheral organs.

### Homing signature profiles of *i*NKT cells from peripheral organs

**Fig. 4:**
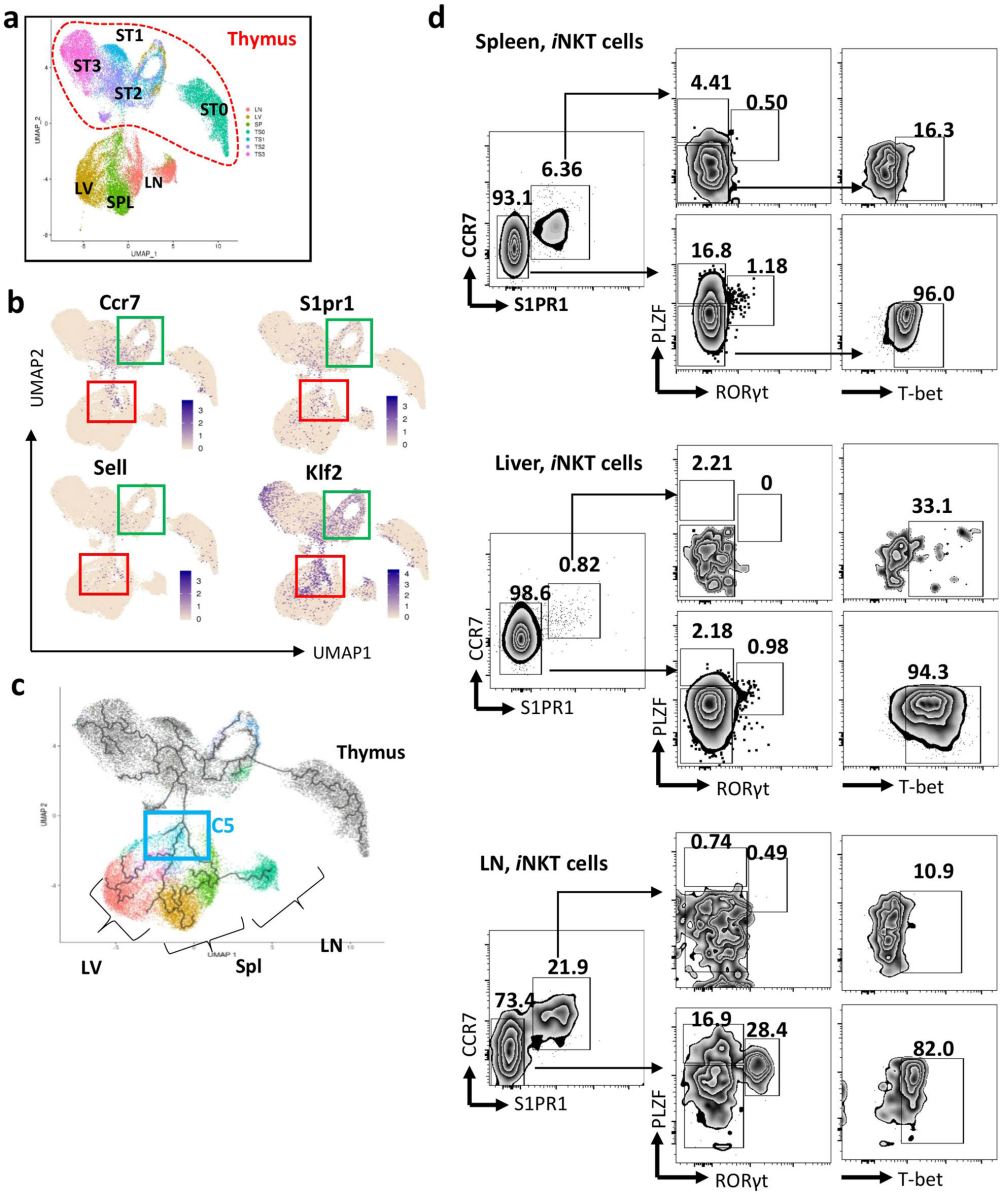
Homing signatures profiles of *i*NKT cells from peripheral organs. **a** UMAP plots showing relationships between *i*NKT cells from different peripheral organs. **b** UMAP plots showing multiple homing markers expression in integrated thymic and peripheral *i*NKT cells. **c** The ordering of *i*NKT cells along pseudotime in a state-space defined by Monocle 3. Each color represents an *i*NKT cluster. **d** Flow cytometry showing RTEs (CCR7^+^S1PR1^+^) and non-RTEs (CCR7^–^S1PR1^−^) from indicated organs. *i*NKT1(PLZF^lo^T-bet^hi^), *i*NKT2 (PLZF^hi^RORγt^−^), and *i*NKT17(PLZF^int^RORγt^+^) cells in RTEs and non-RTEs from indicated organs.

*i*NKT cells exhibit altered patterns of tissue localization, suggesting differences in the signals regulating homing and homeostasis. To directly investigate the *i*NKT cell migration and redistribution mechanism, we integrated different developmental stages of thymic *i*NKT cells with peripheral *i*NKT cell (Fig. 4a). We found that cluster C5 *i*NKT cells from the peripheral organs included candidates for newly arrived *i*NKT cells that share similarities with stage 1/2 thymic *i*NKT cell precursors and express high levels of *Ccr7*^15^. Therefore, this cluster was annotated as an RTE cluster. Consistently, we found multiple homing markers showing similar pseudotime trajectories with *Ccr7*, including *S1pr1*, *Sell*, and *Klf2* (Fig. 4b). In addition, pseudotime analysis carried out using Monocle 3^18–20^ showed three trajectories branching from this intermediate RTE cluster toward terminally differentiated peripheral *i*NKT cells (Fig. 4c). These RTEs are the youngest *i*NKT cells, showing less differentiation/maturity (Fig. 2c). Consistently, we observed that T-bet^+^ *i*NKT1, PLZF^hi^ *i*NKT2, and RORγt^+^ *i*NKT17 cells were substantially underrepresented amongst RTE *i*NKT cells (CCR7^+^ S1PR1^+^) compared to non-RTE *i*NKT cells (CCR7^−^S1PR1^−^) (Fig. 4d).

**Fig. 5:**
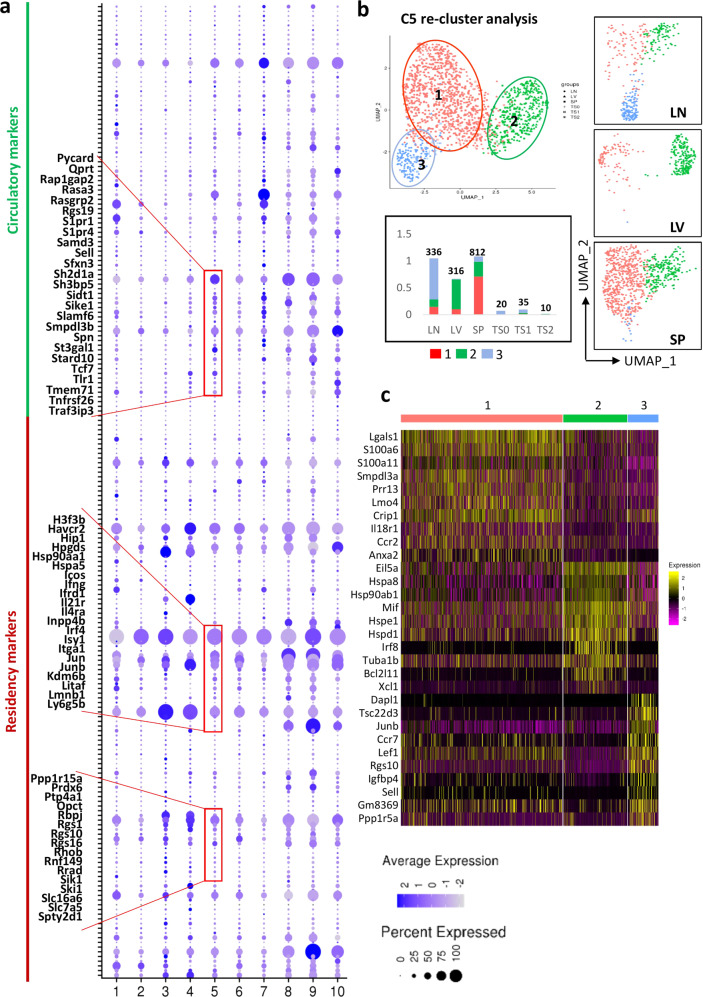
RTEs characters in peripheral *i*NKT cells. **a** Dot plots showing circulatory signatures and residency signature expression in the 10 clusters identified in peripheral *i*NKT cells. **b** UMAP plots showing re-cluster analysis of C5_RTEs from Fig. 1d (top). Bar graph showing the fraction of individual clusters occupied in *i*NKT cells from peripheral and thymus (Stage 0, 1, 2). **c** Heatmap of the top ten differentially regulated genes from each cluster derived from **b**. Each column represents gene expression for an individual cell with color coded on gene expression profiles. Yellow is upregulated and purple is downregulated.

To further describe the *i*NKT cell migration and relocation properties, we characterized the basis of tissue residency of different subsets in spleen, liver, and lymph node by evaluating the expression of the core circulatory and tissue-resident signatures that were reported in a recent study^21^, on the basis of effector CD8 T cells. A gradient was observed in RTEs with an increased circulatory signature (including *Klf2*, *Sell, S1pr1*, and *S1pr4*) and a decrease of tissue residency signatures (including *Atf3*, *Cd244*, *Cd69*, *Fos*, and *Jun*) (Fig. 5a). As RTEs developed and matured in peripheral organs, they lost circulatory signatures, but obtained residency signatures, except cluster C7, which expressed a relative increase in expression of *Klf2*, *S1pr1*, *Klrb1c*, *Bin2*, and *Fam65b* (Fig. 5a). We also found that clusters C3 and C4 of *i*NKT cells from lymph node expressed high levels of most of the tissue residency signature genes, including markers of direct TCR activation (*Jun, Fos, Junb, Jund, Dnala1, Dnala4, Dusp1, Icos, Ppp1r15a, Prdx6, Ptp4a1, Opct, Tnfaip3,* and *Zfp36l1*) (Fig. 5a; Supplementary Fig. S6a). *i*NKT cells from liver and spleen exhibited a higher circulatory property than *i*NKT cells in lymph node, and this pattern is more pronounced in liver (Fig. 5a).

To understand the preferential localization of *i*NKT cells in peripheral organs, we re-clustered RTEs, and three sub-clusters were further identified: C5_1, C5_2, and C5_3 (Fig. 5b, c). Among these sub-clusters, we found the following: spleen RTEs were mainly located in C5_1, expressing high levels of *Lgas1* and *S100a6*, liver RTEs were mainly assigned in C5_2 with high expression of *Irf8* and *Xcl1*, and lymph node RTEs were mainly located in C5_3, expressing high levels of *Dapl1*, *Sell*, and *Tsc22d3*. These data indicated that RTEs, those newly migrated out from thymus, exhibit a greatly different transcriptomic profile. Interestingly, some immature *i*NKT cells from the thymus were clustered together with lymph node cells in C5_3. The great proximity in a UMAP plot highlighted the similar transcriptional expression program between thymic stage 1/2 *i*NKT cells and lymph node RTEs. More precisely, we found that RTEs in lymph node show higher levels of *Ccr7* and *Sell*, but lower *Zbtb16* and *Tbx21* expression levels relative to stage 1/2 *i*NKT cells. In contrast, RTEs from spleen and liver showed a clear differentiation potential, as judged by higher levels of *i*NKT subsets signatures (Supplementary Fig. S6b).

### *Klf2* regulates *i*NKT cell migration and differentiation

**Fig. 6:**
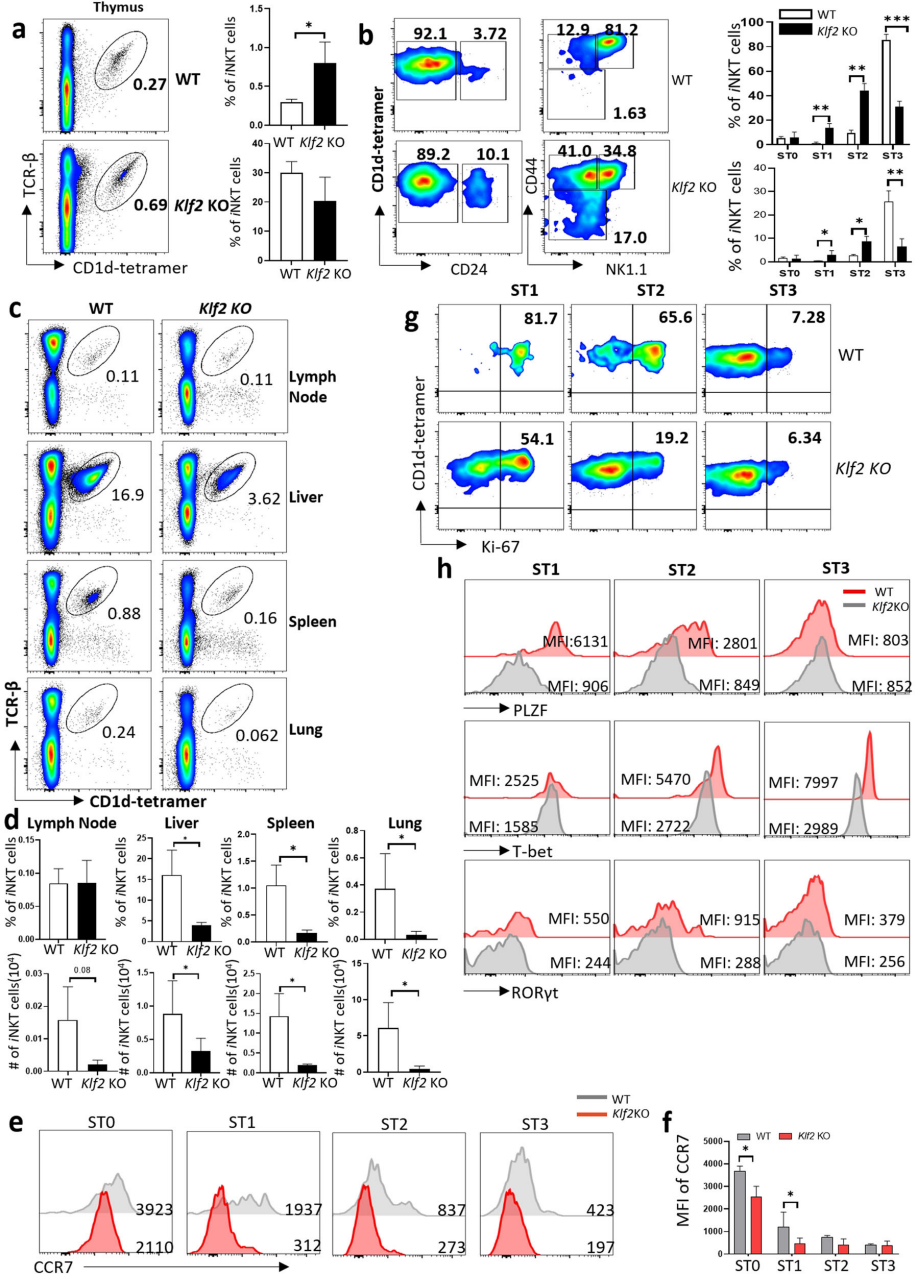
*Klf2* regulates *i*NKT cell migration and differentiation. **a** Representative flow plots of thymic *i*NKT cells from *Klf2* KO and WT mice (left); bar graphs represent means ± SD of frequency and cell number (right), *n* = 3 for *Klf2* KO and WT controls. Data represent two independent experiments. **b** Representative flow plots of different stages (stage 0: CD24^+^; stage 1: CD44^lo^ NK1.1^−^; stage 2: CD44^hi^NK1.1^−^; stage 3: CD44^hi^NK1.1^+^) of *i*NKT from *Klf2* KO and WT mice (left); bar graphs represent means ± SD of frequencies and cell numbers of different developmental stages of *i*NKT cells in *Klf2* KO and WT controls (right). **c**, **d** Representative flow plots of peripheral *i*NKT cells from *Klf2* KO and WT mice. Bar graphs represent means ± SD of frequencies and cells numbers of *i*NKT cells in indicated organs from *Klf2* KO and WT controls. Data represent two independent experiments. **e** Histogram showing CCR7 expression in thymic *i*NKT cell from WT and *Klf2* KO mice. **f** Bar graph showing mean fluorescence intensity (MFI) of CCR7 in *i*NKT cells from WT and *Klf2* KO mice. **g** Representative flow plots showing Ki-67 expression in different developmental stages of thymic *i*NKT cell from WT and *Klf2* KO mice. **h** Histogram showing PLZF, T-bet, and RORγt expression in different developmental stages of thymic *i*NKT cell from WT and *Klf2* KO mice.

The migration patterns of *i*NKT cells are associated with the expression of distinct chemokine receptors, but the underlying molecular mechanism for this regulation is unknown. Transcription factor *Klf2* has been reported to regulate the migration of conventional αβT cells and γδT cells by restricting chemokine receptor expression patterns^22–24^. In our study, *Klf2* was found to be the top gene that was most positively associated with *Ccr7* in RTEs (C5). To test the potential role of *Klf2* in *i*NKT cell development and migration, we examined *i*NKT cell subsets in Lck-Cre *Klf2* deletion mice. We first examined the frequency of *i*NKT cells in the thymus from 6- to 8-week-old *Klf2* KO and WT mice by flow cytometry. As shown in Fig. 6a, *Klf2* KO mice showed a significantly increased frequency of *i*NKT cells in the thymus compared to WT, even though the absolute number are comparable. Further analysis on developmental stages showed higher frequencies and absolute numbers of stage 0, 1, and 2 *i*NKT cells in *Klf2* KO mice (Fig. 6b). In contrast, stage 3 *i*NKT cell frequency and absolute number were significantly reduced in *Klf2* KO mice. These data suggest that deletion of *Klf2* blocked *i*NKT cell development prior to terminal stage 3 maturation.

Given that immature *i*NKT cells with migration potential were blocked and accumulated in the thymus of mice with *Klf2* deletion (Fig. 6b), we measured *i*NKT population in peripheral organs. Consistent with a defect in *i*NKT cell migration from the thymus, the frequency and absolute numbers of *i*NKT cells in peripheral organs, including spleen, liver, and lung, were dramatically diminished in *Klf2* KO mice as compared with WT controls, with the notable exception of the lymph nodes (Fig. 6c, d). These data indicated that *Klf2* mediates *i*NKT cell migration into peripheral organs, especially for spleen, liver, and lung. Evidence to date supports *Klf2* controlling T cell migration by directly regulating the cell surface receptors S1P1 and CCR7^23,25^. To test whether *Klf2* controls *i*NKT cells emigration using a similar mechanism, we measured CCR7 and S1PR1 expression patterns in different developmental stages of *i*NKT cells from *Klf2* KO and WT. As shown in Fig. 6e, f, CCR7 was consistently reduced in immature stages of *i*NKT cell from *Klf2* KO mice. Interestingly, S1PR1 expression in *Klf2* KO mice was consistently reduced at stage 1/2 of iNKT cell development but not at terminally differentiated stage 3 (Supplementary Fig. S7a). Taken together, these data suggest that *Klf2* promotes thymic egress of stage 1/2 *i*NKT cells by regulating homing receptor patterns.

Normally, *i*NKT cells proliferate and expand briskly in stages 1/2. To test whether *Klf2* influenced the proliferating capability of *i*NKT cells, we examined the rate of Ki-67 expression in both *Klf2* KO and WT cells. Compared to WT, *Klf2* KO *i*NKT cells exhibited a lower proliferation status at different developmental stages in the thymus (Fig. 6g). Interestingly, we observed that during development from stage 1 to stage 3, *Klf2* KO *i*NKT cells expressed lower levels of PLZF, T-bet, and RORγt, the master regulators for *i*NKT1/2/17 differentiation (Fig. 6h), and they showed poor differentiation potential (Supplementary Fig. S7b), indicating that *Klf2* is required for *i*NKT cell differentiation. Therefore, not only contributing to tissue localization, *Klf2* may also play important roles in regulating *i*NKT cell effector differentiation.

## Discussion

In this study, we systematically analyzed *i*NKT cell transcriptomic features in various peripheral organs including liver, spleen, and lymph node. A total of ten distinct clusters were identified in integrated peripheral *i*NKT cells, and *i*NKT cells from different peripheral organs showed great phenotypic and functional differences, especially *i*NKT1 cells. In addition, we identified a merged undifferentiated RTE *i*NKT cluster with high *Klf2* expression. More importantly, *Klf2* was further recognized as an essential regulator in *i*NKT migration and differentiation.

Previous studies highlighted a great level of complexity in thymic *i*NKT cells and presented a model of *i*NKT cell development. By integrating scRNA-seq of thymic *i*NKT cells and peripheral *i*NKT cells, we found that thymic *i*NKT cells and peripheral *i*NKT cells are clearly separated in UMAP plot, with a small RTE cluster connecting them.

Peripheral *i*NKT cells exhibited substantial heterogeneity in their degree of maturation. In contrast to thymic *i*NKT cells, which require CD1d for their initial selection and maturation, *i*NKT cells do not require continual CD1d interactions in the periphery to support homeostatic proliferation, long-term survival, or to maintain tissue distribution^26^. However, *i*NKT cells require several chemokine receptors and integrins for their maintenance in the specific peripheral organs and these interactions between *i*NKT cells and the microenvironment help generate organ-specific *i*NKT populations. For example, *i*NKT cells located in the spleen partially require *Cxcl13*; however, *Cxcl13* is not required for *i*NKT cells in liver. Meanwhile, *i*NKT cells that home to the liver require *Cxcr3* and *Itgβ2*, yet these receptors do not contribute to splenic migration^4,27,28^. Even though *i*NKT cells have a restricted TCR profile, there were functional differences from organ to organ. Previous reports have shown that *i*NKT cells from the liver and spleen have different anti-tumor activities^14^. More precisely, *i*NKT cells from liver, including CD4^+^ *i*NKT cells and CD4^–^ *i*NKT cells were better able to reject tumor cells than their counterparts from the spleen or thymus. However, the organ-specific mechanisms for *i*NKT cells functional differences are still unclear^9^. In our study, we revealed that transcriptome profiles of *i*NKT cells were organ-specific, especially for *i*NKT1 cells. There was a marked phenotypical difference between thymic *i*NKT1 cells and peripheral *i*NKT1 cells, even among peripheral organs, *i*NKT1 cells also exhibited a significant difference. *i*NKT cells from liver and spleen exhibited a higher circulatory property than *i*NKT cells in lymph node, and this pattern is more pronounced in liver. In addition, *i*NKT1 cells in lymph node showed great TCR activation properties. These differences should reflect: (1) anatomical tissue structure, e.g., *i*NKT cells locate at the parenchyma in spleen, but locate at vasculature in liver^29^; (2) conventional microbiota in local environment and specific antigenic stimulation in the indicated organs^30^; and (3) organ-specific antigen-presenting cells (APCs) could also contribute to *i*NKT cell function and phenotype in local environments. Previous studies suggested that different levels of costimulatory molecules on organ-specific APCs cause different *i*NKT cell responses. APCs in different organs may also express different tissue-specific glycolipid ligands for *i*NKT cells^31^. For example, APCs in the liver might capture and present exogenous glycolipids from the alimentary tract, which could promote IFN-γ-dependent functions^32,33^. However, unlike *i*NKT1 cells, which are organ-specific, *i*NKT17 cells are very similar transcription-wise in different peripheral organs, indicating that *Rorc* is the major determinant for the *i*NKT17 cell phenotype.

*i*NKT cells first exit the thymus in a phenotypically and functionally immature state and require a period of post-thymic maturation before transitioning into the mature/effector *i*NKT cell compartment. Here, we found that the transcriptional program of RTEs was very different from those of mature *i*NKT cells in spleen, liver, and lymph node. For example, (1) RTEs in peripheral organs are not well differentiated; (2) RTEs in peripheral organs exhibit a higher proliferating activity; (3) RTEs in peripheral organs exhibit pronounced circulatory properties; (4) RTEs in peripheral organs express high level of homing signatures, including transcription factor *Klf2*. Previous reports have shown that *Klf2* transactivates *S1p1r*, *Ccr7*, and *Sell* promoters, and that deletion of *Klf2* leads to accumulation of CD4^+^ and CD8^+^ αβT cells in the thymus and preferential homing to peripheral tissues^24,25^. In this study, we found that *Klf2* was mainly expressed by stage 1 and 2 *i*NKT cells in the thymus and RTEs in peripheral tissues. Loss of *Klf2* led to a defect in *i*NKT cell emigration; a result that is supported by previous studies^15^. Our scRNA-seq study also offered an exciting opportunity for mapping peripheral RTEs. Pseudotime analysis showed that *Klf2* and *Ccr7* have a similar expression trajectory and they had a fine correlation during *i*NKT cell development. Deletion of *Klf2* showed severely impaired *i*NKT cell differentiation and migration. Consistent with this finding, *Ccr7* expression was significantly reduced in *Klf2*-deficient *i*NKT cells. A previous report indicated that deletion of *Klf2* mediated by CD4Cre caused increased expansion or survival of thymic PLZF^+^ T cells^34^, and therefore promoted memory-like phenotype (CD44^hi^, CD122^hi^) CD8 T cells. However, expressing PLZF^+^ T cells in mouse thymus may include *i*NKT cell, γδNKT cells, and MAIT cells, *Klf2* function in *i*NKT cell development were not fully explored. In our current study, we used LckCre *Klf2* KO mice, in which *Klf2* was deleted in the early stage of T cells development. We found that the frequency of *i*NKT cells was increased in thymus from *Klf2*-deficient mice, and most of these *i*NKT cells were blocked at immature stages (stage 1 and stage 2). Interestingly, peripheral *i*NKT cells from Lck Cre *Klf2* KO mice were significantly reduced. Integrating analysis with scRNA-seq data suggested that *Klf2* regulate *i*NKT cells migration, and deletion of *Klf2* blocked thymic *i*NKT cells outward migration. Therefore, our study further expands previous study and explained the mechanism of *Klf2* in regulation thymic *i*NKT cells.

In addition, it is important to mention that extreme cytotoxic cluster C7, with high levels of *Gzma* and *Ccl5* expression, also exhibit a high expression level of *Klf2* and other circulatory signatures but fewer residency signatures. Cluster C7 and C5_RTE clusters showed similar expression programs, judged by their proximity in UMAP plots. We therefore assumed that cluster C7 could be relatively new *i*NKT cells that directly migrate from thymus, rather than long-term *i*NKT cells that are from super mature *i*NKT1 cells. However, their precise developmental trajectory is still unclear. In addition, it is not clear whether specific organ targeting is determined in the thymus by distinct patterns of integrin and chemokine receptor expression or if migration to a given tissue is stochastic, only dependent on subset-specific niches. More important, even though our study revealed the phenotypic differences of *i*NKT cells in different peripheral organs, we are still unclear about how these phenotypic differences contribute to varying function in different disease models.

In summary, we demonstrate here that *i*NKT cells from liver, spleen, and lymph node appear to have distinct phenotypic profiles and functional capabilities, especially for *i*NKT1 cells. Meanwhile, we identified *Klf2* in peripheral RTEs as playing a critical role in *i*NKT cell differentiation and migration.

## Materials and methods

### Mice

C57BL/6 were purchased from Jackson Laboratory (Bar Harbor, ME). Lck-Cre *Klf2* KO mice were provided by Prof. Eric Sebzda (Wayne University, Detroit, MI). Briefly, mice carrying a floxed allele of *Klf2* (*Klf2*^fl/fl^)^35^ were mated to transgenic C57BL/6 mice expressing Cre recombinase under the guidance of a proximal *Lck* promoter (obtained from the Jackson Laboratory), to generate Lck*-cre;Klf2*^fl/fl^ conditional knockout mice (*Klf2* KO). *i*NKT cells from peripheral organs from 5-week-old C57BL/6 mice were utilized for scRNA-seq study; 6–8-week-old, sex-matched mice were utilized for *Klf2* function studies. All studies, protocols, and mouse handling were approved by the Institutional Animal Care and Use Committee.

### Flow cytometry gating strategy and antibodies

Single-cell suspensions were washed twice with FACS staining buffer (1× PBS, 2% FBS) and incubated with Fc block (clone 2.4G2). Cells were stained with anti-mouse PBS57-loaded CD1d-tetramer (provided by the NIH Tetramer Core Facility). The following fluorescence conjugated antibodies were used: anti-TCRβ (H57-597), anti-CD24 (M1/69), anti-CD44 (IM7), anti-NK1.1 (PK136), anti-RORγt (B2D), anti-PLZF (Mags.21F7), anti-T-bet (eBio4B10 (4B10)), anti-Ki-67 (520914), anti-CCR7 (4B12), anti-S1PR1 (JM10-66), anti-IFIT1 (OTI3G8), anti-Aqp3 and anti-Gzma (GzA-3G8.5). Cell surface staining was performed with staining buffer; intranuclear staining was performed with eBioscience Fixation/permeabilization buffer. The flow cytometry assay was performed through BD FACSCelesta and data were analyzed using FlowJo V10.2 software. Gating strategy: after gating on lymphocyte, doublets were excluded by using forward scatter (FSC) and side scatter (SSC), mouse *i*NKT cells were further identified as TCRβ^+^ CD1d-tetramer^+^.

### Mouse iNKT cell enrichment and sorting

Mouse *i*NKT cell enrichment and sorting strategy was described previously^36^. Briefly, peripheral organs from mouse, including spleen, liver, and lymph nodes were harvested from 5-week-old C57BL/6 mice. For the enrichment of *i*NKT cells, total cells were stained with biotin-conjugated anti-mouse CD8 Ab, anti-mouse B220 Ab, and anti-biotin magnetic beads (eBioscience). Negatively selected CD8^−^ and B220^−^ cells were then stained with anti-mouse TCRβ, CD1d-tetramer Abs. *i*NKT cells of whole population were further sorted from C57BL/6 mouse spleen, lymph node, and liver using FACSAria II Usage, cells collected with purity > 97%.

### scRNA-seq library generation

scRNA-seq library generation was described in our previous published study^37^. Two biological repeats for each samples (including *i*NKT cells from spleen, liver, and lymph node) of scRNA-seq libraries were generated using the 10X Genomics Chromium Single Cell 3′ Reagent Kit (v2 Chemistry) and Chromium Single Cell Controller as previously described^38^.

### scRNA-seq data analysis

Sequence reads from scRNA-seq libraries were demultiplexed and aligned to the mm10 mouse reference, barcode processed, and UMI counted using the 10X Genomics Cell Ranger (V3.1.0) pipeline^38^. Estimated number of cells captured per sample was between 2646 and 2867 with 60,622–70,044 mean reads per cell, 1788–1388 median genes per cell, and 1468–3597 median UMI counts per cell. A total of 16,587 cells with 2179 UMI counts/cell in average were selected via Cell Ranger for further analysis for all of six samples. Datasets were subsequently analyzed using the R Seurat package^39,40^. Principle Component Analysis (PCA) was employed to analyze combined samples. Quality control metrics employed are as follows. We employed two strategies to identify potential doublets. First, cells expressing both Xist and Y chromosome genes (*Kdm5d*, *Eif2s3y*, *Gm29650*, *Uty*, and *Ddx3y*) were excluded from the dataset. Second, cells expressing uncharacteristically high numbers of genes (> 4000) were excluded. Low-quality cells were excluded based on a low number of genes detected (<300) and/or having high mitochondrial genetic content (> 15%). A total of 14,986 genes in 15,317 cells passed these quality control measures. Genes removed include ribosomal structural proteins (as identified by gene ontology term GO: 0003735 and the Ribosomal Protein Gene (RPG) database 4), non-coding rRNAs, Hbb, and genes not expressed in ≥ 3 cells. A total of 14,986 genes in 15,317 cells passed these quality control measures.

A global-scaling normalization method “LogNormalize” in Seurat was employed to normalize gene expression measurements of each cell by the total expression, multiplying this by a factor of 10,000, followed by log-transformation. Highly variable genes in each data analysis were identified, and the intersecting top 3000 genes in each dataset were used for clustering and downstream analyses. Datasets underwent scaling and regressing on the number of detected molecules per cell (nUMI) and the percentage of mitochondrial gene content (pct.mito). The number of principal components (PCs) used to cluster cells was determined by manual inspection of the scree plot. After identifying the number of PCs to be included for downstream analyses (20 PCs), a graph-based clustering approach implemented in Seurat was used to iteratively cluster cells into groups, based on similarities of those components among cells. The UMAP method was utilized to visualize resulting clusters. To assess the effects of cell cycle heterogeneity, cell cycle phase scores (G2/M and S phases) were calculated based on canonical markers and used to regress out the data^41^. The FindAllMarkers function in Seurat was then implemented to identify differentially expressed genes between clusters with a fold-change of >2 and a Bonferroni adjustment of *P* value < 0.05 as a statistical significance threshold. To determine if differentially expressed genes belong to identifiable groups, pathway analysis was carried out using the Ingenuity Pathway Analysis (IPA, Qiagen Bioinformatics, Redwood City, CA).

### Statistical analysis

For comparison between groups, statistical analysis was performed by unpaired *t* test with GraphPad Prism 8.0.

## Supplementary information

Supplementary Information

## Data Availability

ScRNA-seq that support the findings of this study have been deposited in the NCBI Gene Expression Omnibus (GEO; http://www.ncbi.nlm.nih.gov/geo/) with the accession numbers GSE130184 and GSE161495, respectively. All relevant data are available from the authors upon reasonable request.

## References

[1] Das R, Sant’Angelo DB, Nichols KE (2010). Transcriptional control of invariant NKT cell development. Immunol. Rev..

[2] Kumar V, Delovitch TL (2014). Different subsets of natural killer T cells may vary in their roles in health and disease. Immunology.

[3] Bendelac A, Savage PB, Teyton L (2007). The biology of NKT cells. Annu. Rev. Immunol..

[4] Thomas SY (2011). PLZF induces an intravascular surveillance program mediated by long-lived LFA-1-ICAM-1 interactions. J. Exp. Med..

[5] Benlagha K, Weiss A, Beavis A, Teyton L, Bendelac A (2000). In vivo identification of glycolipid antigen-specific T cells using fluorescent CD1d tetramers. J. Exp. Med..

[6] Hammond KJ (2001). CD1d-restricted NKT cells: an interstrain comparison. J. Immunol..

[7] Matsuda JL (2000). Tracking the response of natural killer T cells to a glycolipid antigen using CD1d tetramers. J. Exp. Med..

[8] Crosby CM, Kronenberg M (2018). Tissue-specific functions of invariant natural killer T cells. Nat. Rev. Immunol..

[9] Seino K, Taniguchi M (2005). Functionally distinct NKT cell subsets and subtypes. J. Exp. Med..

[10] Slauenwhite D, Johnston B (2015). Regulation of NKT cell localization in homeostasis and infection. Front Immunol..

[11] Lee YJ (2015). Tissue-specific distribution of iNKT cells impacts their cytokine response. Immunity.

[12] Torina A, Guggino G, La Manna MP, Sireci G (2018). The Janus face of NKT cell function in autoimmunity and infectious diseases. Int. J. Mol. Sci..

[13] Berzins SP, Smyth MJ, Baxter AG (2011). Presumed guilty: natural killer T cell defects and human disease. Nat. Rev. Immunol..

[14] Crowe NY (2005). Differential antitumor immunity mediated by NKT cell subsets in vivo. J. Exp. Med..

[15] Wang H, Hogquist KA (2018). CCR7 defines a precursor for murine iNKT cells in thymus and periphery. Elife.

[16] Engel I (2016). Innate-like functions of natural killer T cell subsets result from highly divergent gene programs. Nat. Immunol..

[17] Barnes SE (2015). T cell-NF-kappaB activation is required for tumor control in vivo. J. Immunother. Cancer.

[18] Qiu X (2017). Reversed graph embedding resolves complex single-cell trajectories. Nat. Methods.

[19] Trapnell C (2014). The dynamics and regulators of cell fate decisions are revealed by pseudotemporal ordering of single cells. Nat. Biotechnol..

[20] Cao J (2019). The single-cell transcriptional landscape of mammalian organogenesis. Nature.

[21] Milner JJ (2017). Runx3 programs CD8(+) T cell residency in non-lymphoid tissues and tumours. Nature.

[22] Odumade OA, Weinreich MA, Jameson SC, Hogquist KA (2010). Kruppel-like factor 2 regulates trafficking and homeostasis of gammadelta T cells. J. Immunol..

[23] Carlson CM (2006). Kruppel-like factor 2 regulates thymocyte and T-cell migration. Nature.

[24] Sebzda E, Zou Z, Lee JS, Wang T, Kahn ML (2008). Transcription factor KLF2 regulates the migration of naive T cells by restricting chemokine receptor expression patterns. Nat. Immunol..

[25] Bai A, Hu H, Yeung M, Chen J (2007). Kruppel-like factor 2 controls T cell trafficking by activating L-selectin (CD62L) and sphingosine-1-phosphate receptor 1 transcription. J. Immunol..

[26] McNab FW (2005). The influence of CD1d in postselection NKT cell maturation and homeostasis. J. Immunol..

[27] Johnston B, Kim CH, Soler D, Emoto M, Butcher EC (2003). Differential chemokine responses and homing patterns of murine TCR alpha beta NKT cell subsets. J. Immunol..

[28] Kim EY, Lynch L, Brennan PJ, Cohen NR, Brenner MB (2015). The transcriptional programs of iNKT cells. Semin. Immunol..

[29] Liew PX, Kubes P (2015). Intravital imaging—dynamic insights into natural killer T cell biology. Front. Immunol..

[30] Mallevaey T, Selvanantham T (2012). Strategy of lipid recognition by invariant natural killer T cells: ‘one for all and all for one’. Immunology.

[31] Yang Y (2003). Control of NKT cell differentiation by tissue-specific microenvironments. J. Immunol..

[32] Mattner J (2005). Exogenous and endogenous glycolipid antigens activate NKT cells during microbial infections. Nature.

[33] Kinjo Y (2005). Recognition of bacterial glycosphingolipids by natural killer T cells. Nature.

[34] Weinreich MA, Odumade OA, Jameson SC, Hogquist KA (2010). T cells expressing the transcription factor PLZF regulate the development of memory-like CD8+ T cells. Nat. Immunol..

[35] Lee JS (2006). Klf2 is an essential regulator of vascular hemodynamic forces in vivo. Dev. Cell.

[36] Wang J (2019). miR-183-96-182 cluster is involved in invariant NKT cell development, maturation, and effector function. J. Immunol..

[37] Zhou L (2020). Single-cell RNA-seq analysis uncovers distinct functional human NKT cell sub-populations in peripheral blood. Front. Cell Dev. Biol..

[38] Zheng GX (2017). Massively parallel digital transcriptional profiling of single cells. Nat. Commun..

[39] Butler A, Hoffman P, Smibert P, Papalexi E, Satija R (2018). Integrating single-cell transcriptomic data across different conditions, technologies, and species. Nat. Biotechnol..

[40] Stuart T (2019). Comprehensive integration of single-cell data. Cell.

[41] Tirosh I (2016). Dissecting the multicellular ecosystem of metastatic melanoma by single-cell RNA-seq. Science.

