# Supplementary material for: Single-cell analysis reveals differences among iNKT cells colonizing peripheral organs and identifies Klf2 as a key gene for iNKT emigration
Source: Cell Discov. 2022 Aug 2;8:75. doi: 10.1038/s41421-022-00432-z (PMC9343440; doi:10.1038/s41421-022-00432-z)
Supplement: Supplementary file 1 — Supplementary Information [file 41421_2022_432_MOESM1_ESM.pdf]

Supplementary Fig. S1

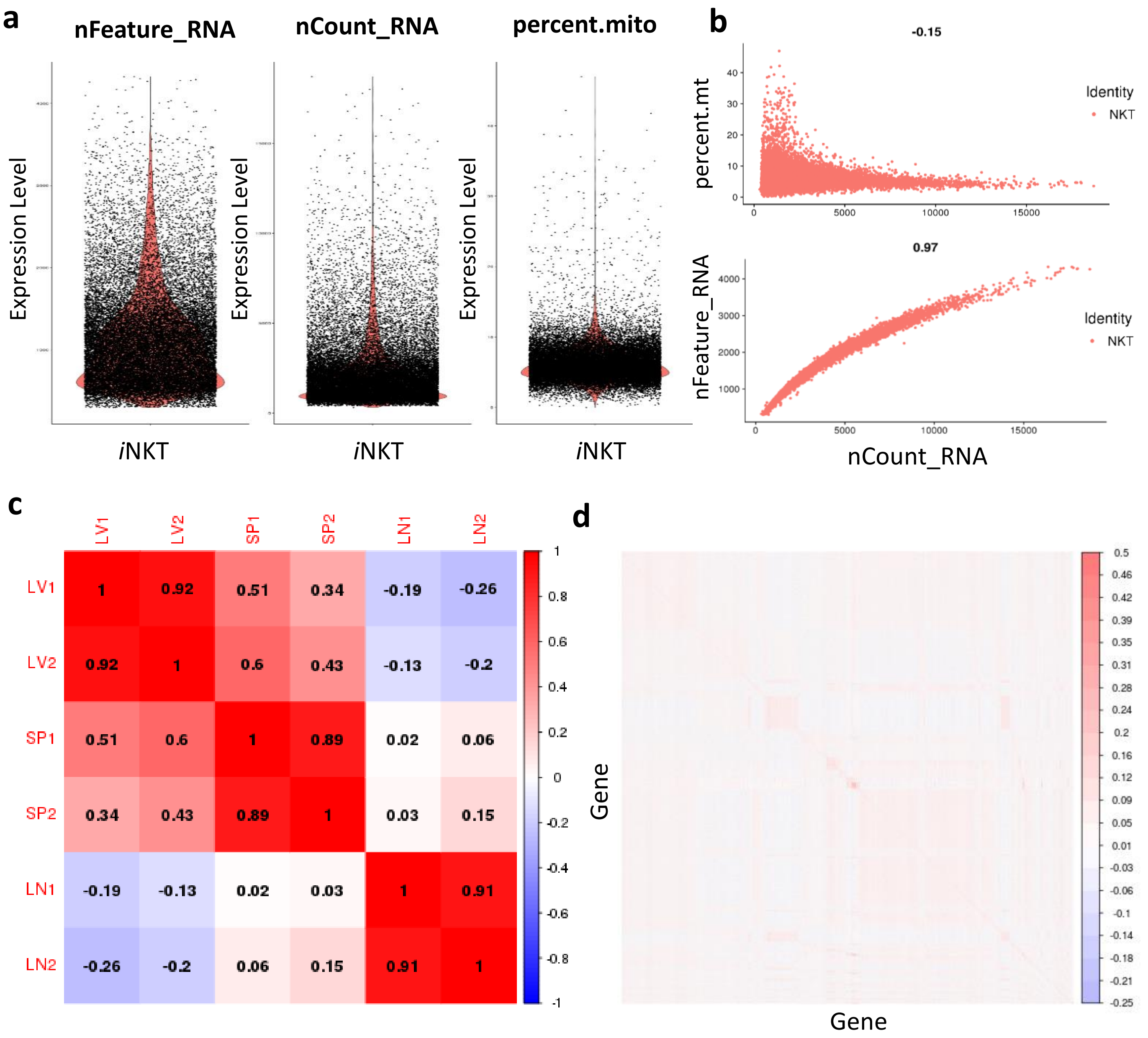

Supplementary Fig. S1: Overview of the cell types in peripheral *i*NKT cells identified by scRNA-seq

a-b. Quality controls for the single cell RNA-seq study.
c. Pearson correlation matrix of the average expression profiles, based upon all differentially expressed genes all duplicated samples analyzed.
d. Pearson correlation matrix of the gene expression profiles.

Supplementary Fig. S2

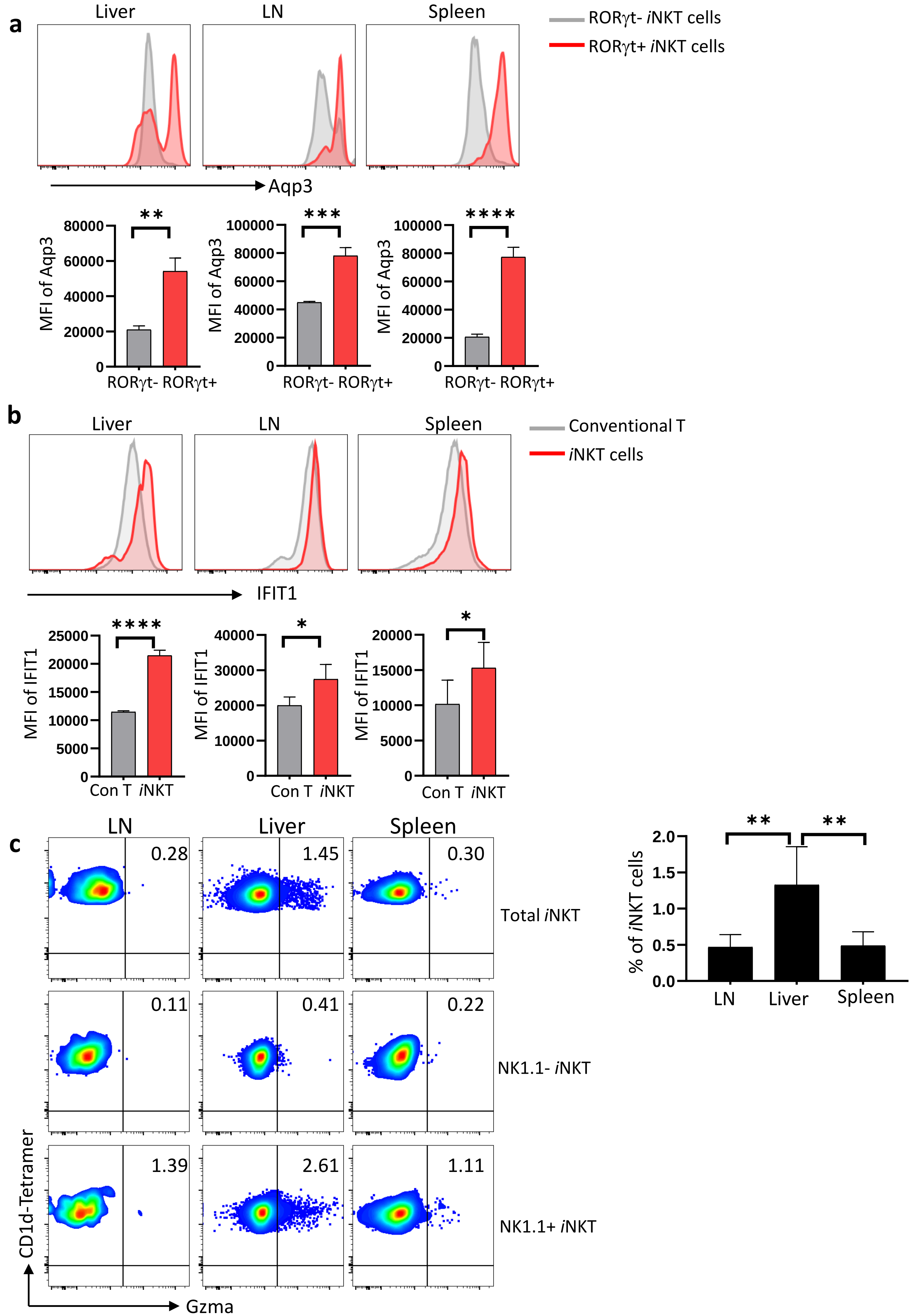

Supplementary Fig. S2: Phenotypic profiles of mouse peripheral *i*NKT cells

a. Histogram showing Aqp3 expression in RORγt- *i*NKT cells (grey) and RORγt+ *i*NKT cells(red). b. Histogram showing IFIT1 expression in conventional T cells(grey) and *i*NKT cells (red). Bar graph showing mean fluorescence intensity (MFI) of IFIT1 expression. c. Flow cytometry plots showing Gzma expression in peripheral *i*NKT cells and their NK1.1- and NK1.1+ subsets. Bar graph showing the frequency of Gzma+ *i*NKT cells in different peripheral *i*NKT cells. Data represent three independent experiments, data were analyzed by a two-sided paired t-test, * P<0.05, ** P<0.01, *** P<0.001, and **** P<0.001 .

Supplementary Fig. S3

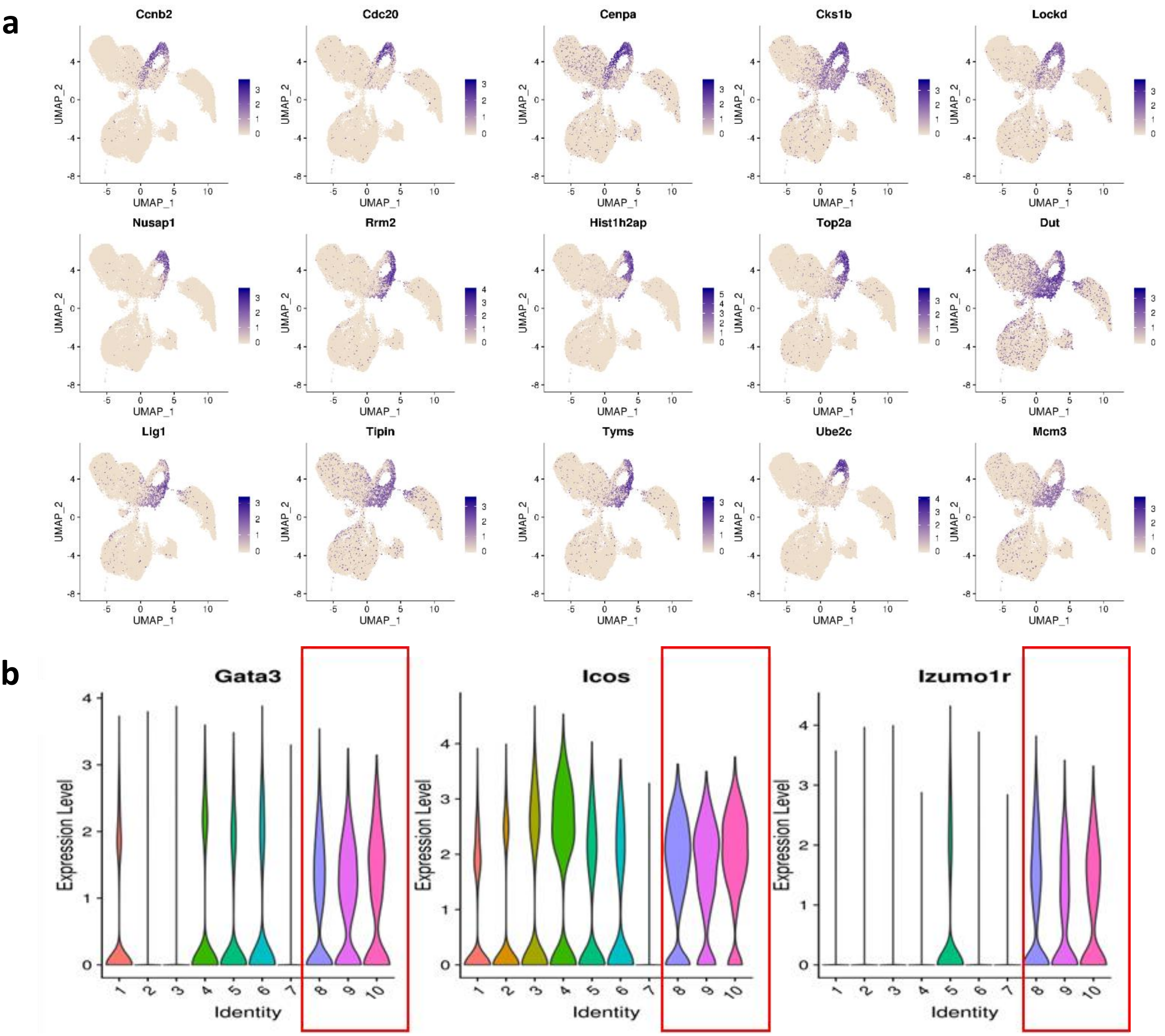

Supplementary Fig. S3: Gene expression patterns in peripheral *i*NKT cells

a. UMAP plots depicting single-cell gene expression trajectory of genes relative to cell cycle in peripheral *i*NKT cell development. b. Violin plots showing that *Gata3*, *Icos*, and *Izumo1r* expression in C1-C10.

Supplementary Fig. S4

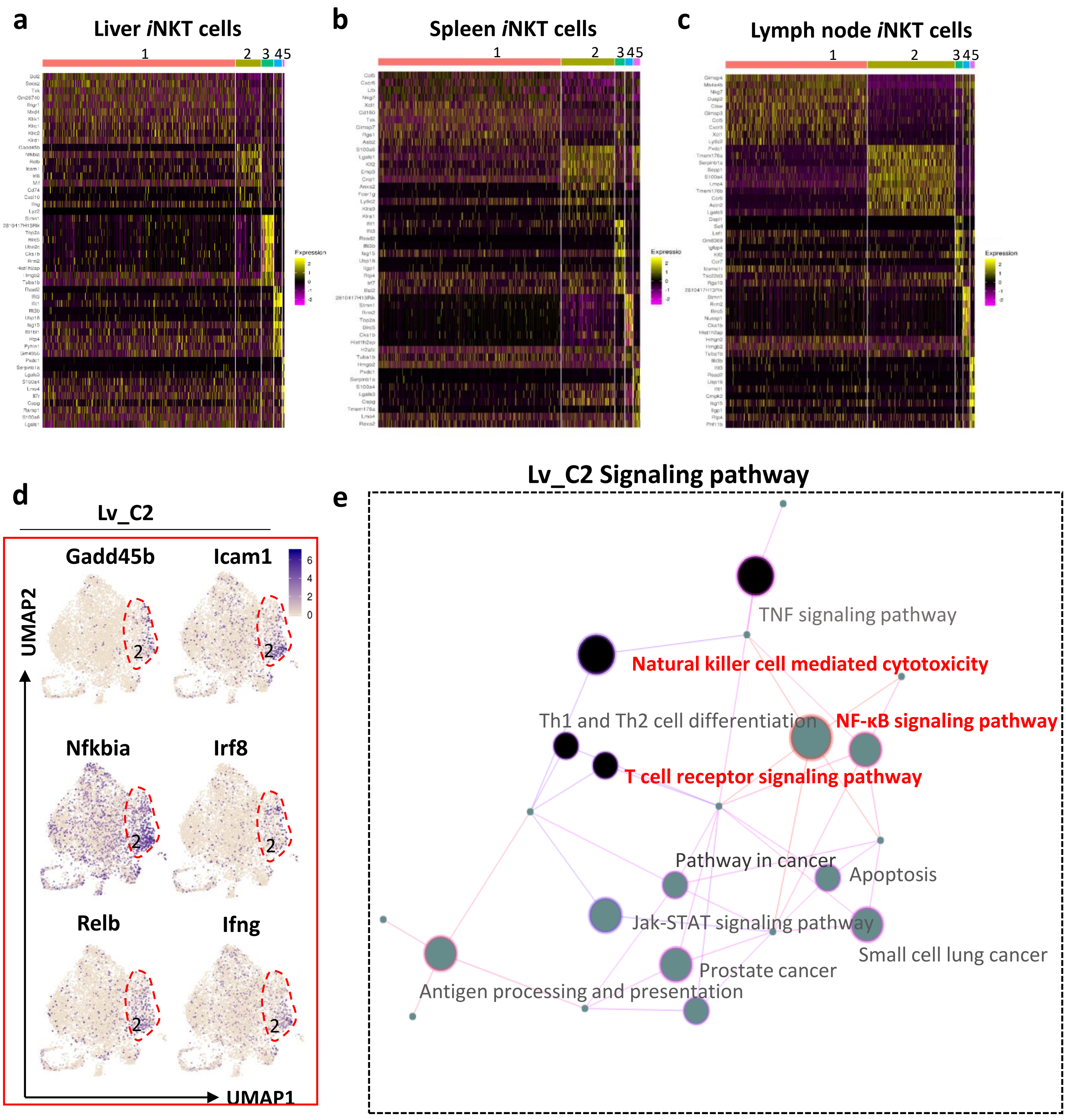

Supplementary Fig. S4: Cluster analysis of *i*NKT cells from different peripheral organs

a-c. Heatmap of the top ten differentially regulated genes from each cluster derived from Fig. 3a(Liver), Fig. 3b (Spleen) and Fig. 3b (lymph node). Each column represents gene expression for an individual cell with color coded on gene expression profiles. Yellow is up and purple is down. d. UMAP plots depicting single-cell genes expression trajectory of *Gadd45b*, *Icam1*, *Nfkbia*, *Irf8*, *Relb*, and *Ifng* in liver *i*NKT cells. e. Signal pathway enriched in cluster 2 of liver *i*NKT cells.

Supplementary Fig. S5

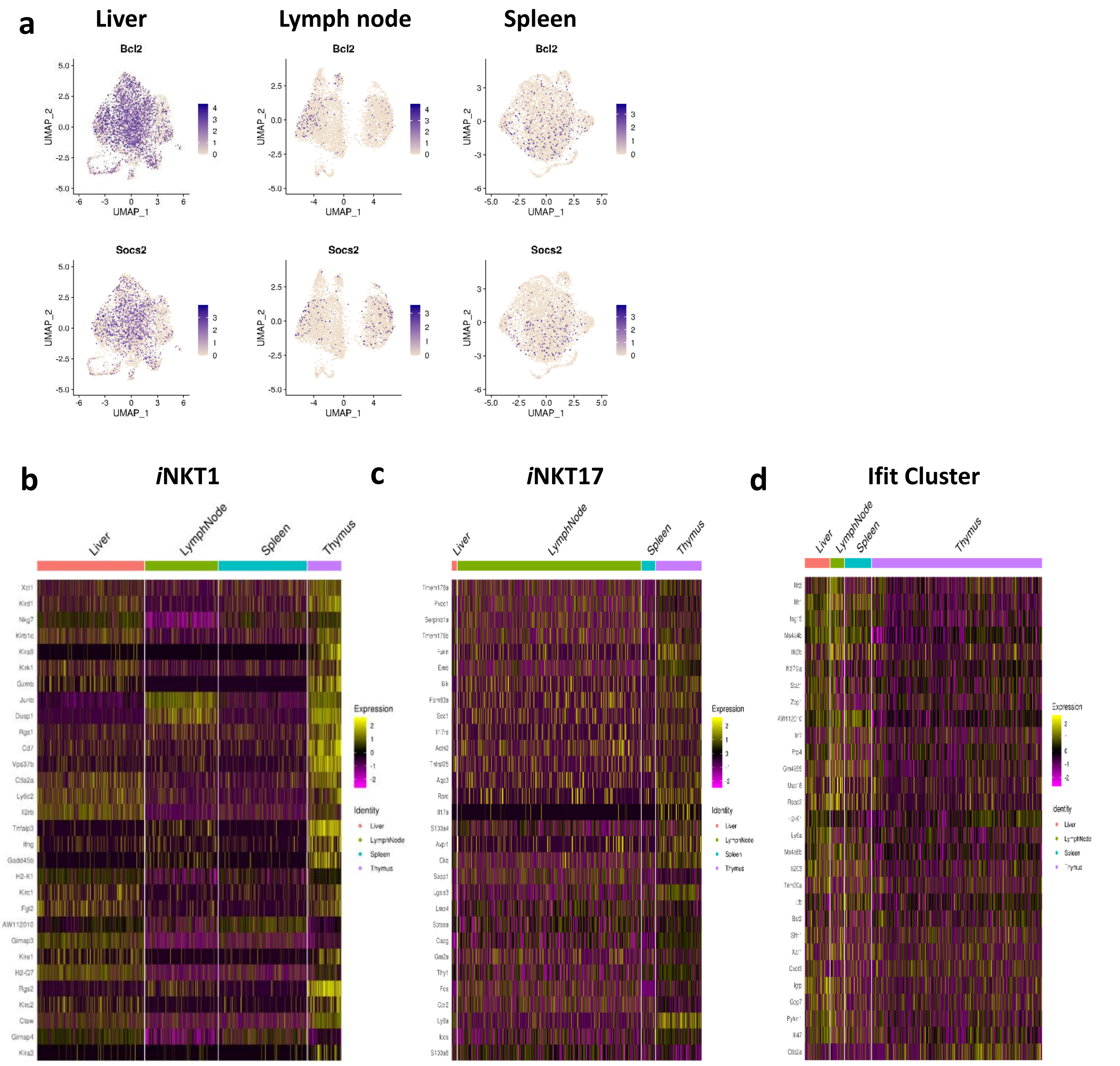

Supplementary Fig. S5: Comparison of transcriptomic profiles among *i*NKT cells from different peripheral organs

a. UMAP plots depicting single-cell genes expression of *Bcl2* and *Socs2* in peripheral *i*NKT cell development. b-d. Heatmap of the top ten differentially regulated genes from indicated cluster derived from thymic and peripheral organs. Each column represents gene expression for an individual cell with color coded on gene expression profiles. Yellow is up and purple is down.

Supplementary Fig. S6

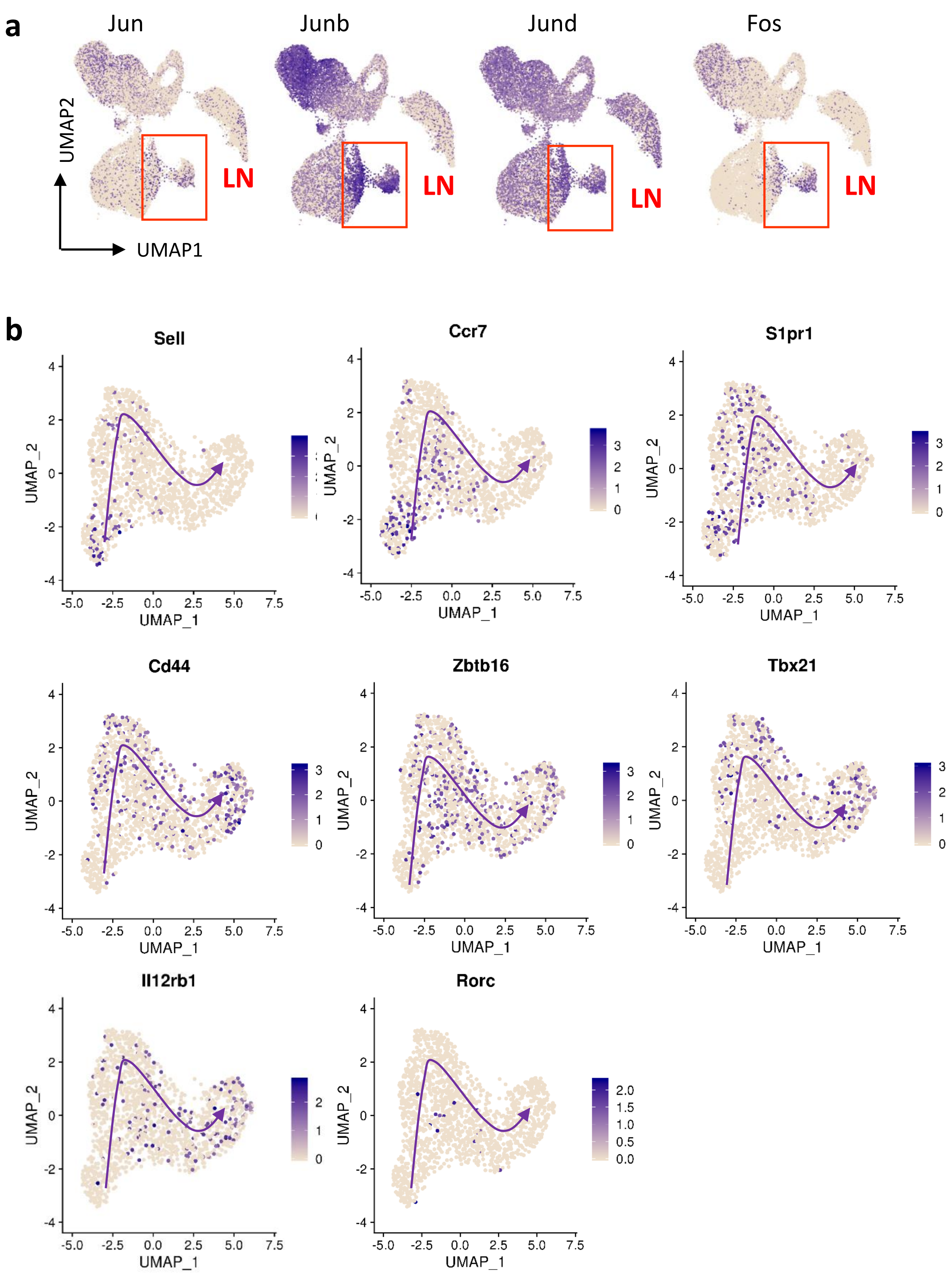

Supplementary Fig. S6: Homing signatures profiles of *i*NKT cells from peripheral organs

a. UMAP plots depicting single-cell genes expression trajectory of T cell activity genes in thymic and peripheral *i*NKT cells.

b. UMAP plots depicting single cell genes expression trajectory in C5_RTEs.

Supplementary Fig. S7

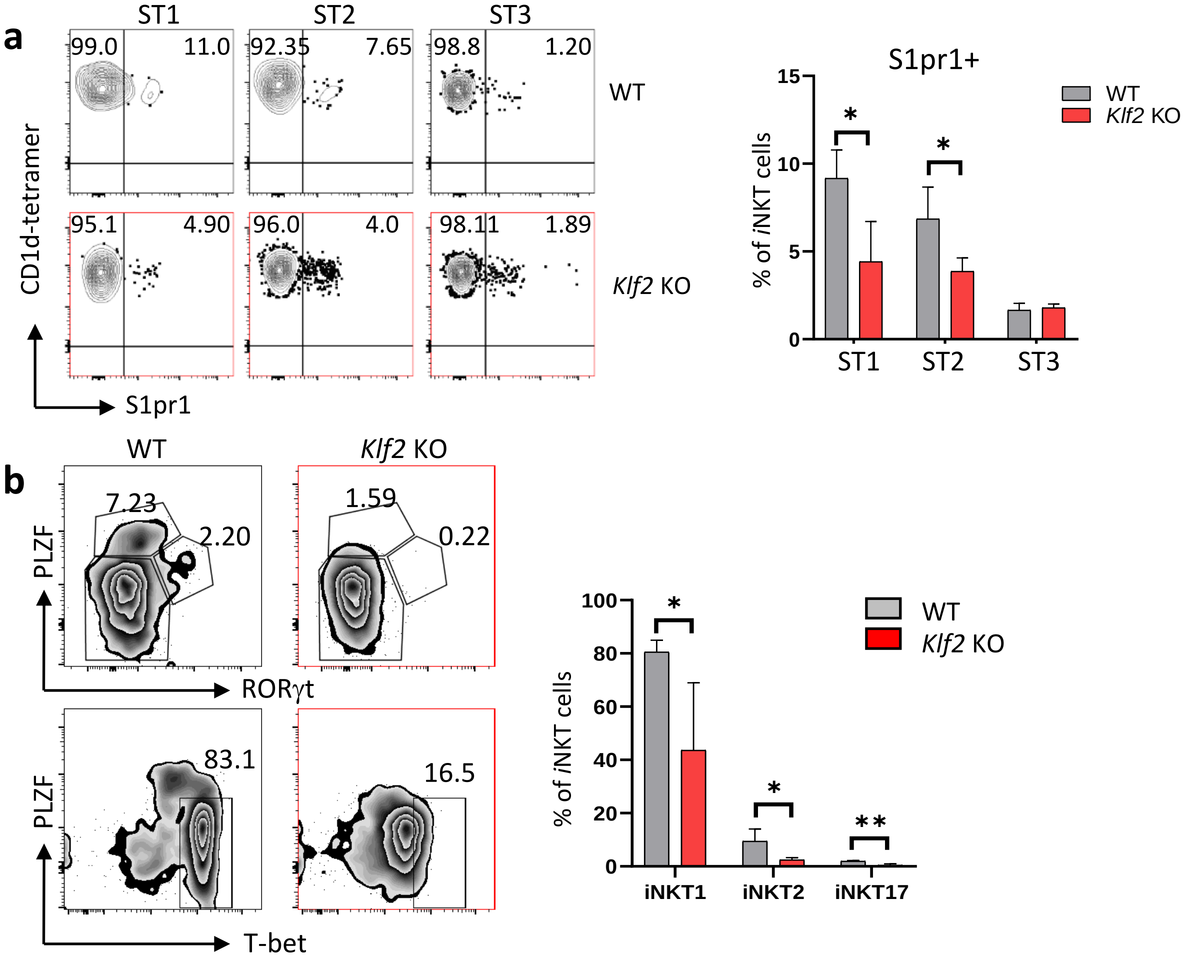

Supplementary Fig. S7: The role of *Klf2* in *i*NKT cells development.

a. S1pr1 expression in different stages (ST1-ST3) of *i*NKT cells from *Klf2* KO and WT controls (left). Bar graphs showing the S1pr1 in indicated stages of *i*NKT cells from *Klf2*KO and WT controls. b. Flow cytometry showing *i*NKT1 (PLZF^{lo} T-bet^{+}), *i*NKT2 (PLZF^{hi} RORγt^{-}), and *i*NKT17 (PLZF^{int} RORγt^{+}) in *i*NKT cells from Klf2 KO and WT controls. Data represent three independent experiments, data were analyzed by a two-sided paired t-test, * *P*<0.05.
